# Beyond membership: how active sports club participation shapes happiness

**DOI:** 10.3389/fpubh.2026.1896287

**Published:** 2026-07-22

**Authors:** Kuntong Wu, Zhengbing Guo, Jing Ren

**Affiliations:** 1 Xi'an Siyuan University, Xi'an, China; 2 School of Humanities and Social Science, Xi’an Jiaotong University, Xi'an, China

**Keywords:** active participation, happiness, leisure-time sports participation, sports club participation, venue-based exercise

## Abstract

**Introduction:**

Happiness has become an important public health and policy concern, and physical activity is increasingly viewed as a relevant lifestyle-related factor. Against this background, this study examines whether the association between sports club participation (SCP) and happiness depends on active participation rather than nominal membership alone. Its contribution is to distinguish formal membership from actual engagement in sports clubs and to examine whether the SCP-happiness association is statistically linked to two behavioral dimensions of physical activity: leisure-time sports participation (LTSP) and venue-based exercise (VBE).

**Methods:**

Using data from the 2023 Chinese General Social Survey, the analysis distinguishes among non-members, inactive members, and active members. Ordinary least squares regression, ordered logit models, propensity score matching, and bootstrap-based parallel mediation analysis were applied to examine the association between SCP and happiness and the statistical indirect pathways through LTSP and VBE.

**Results:**

The results show that active SCP is positively associated with happiness (*β* = 0.151, *p* < 0.001), whereas inactive membership is not. After LTSP and VBE are introduced, the coefficient for active SCP declines but remains significant (*β* = 0.098, *p* < 0.05). Both LTSP and VBE are positively associated with happiness and statistically mediate the association between active SCP and happiness.

**Discussion:**

These findings suggest that the relevance of sports clubs for happiness is not attached to formal membership alone, but is mainly observed when membership involves active engagement and greater participation in physical activity. Given the cross-sectional design, the findings should be interpreted as associational rather than causal evidence.

## 1 Introduction

Happiness has become increasingly important outcomes in research on quality of life and public health. A large body of scholarship has shown that physical activity is positively associated with mental health, happiness, and broader social outcomes (1). In the Chinese context, recent evidence likewise suggests that physical activity is positively related to happiness (2), while newer studies indicate that leisure-time physical activity may be especially beneficial for happiness and mood (3). Yet much of this literature still approaches physical activity primarily as an individual behavior, emphasizing what exercise does for the body and the mind, rather than asking how different social forms of participation may shape these benefits.

This question matters because, in contemporary society, sport is increasingly practiced not only as a private or spontaneous activity but also as an organized social practice. Sports clubs are one of the clearest manifestations of this transformation (4). They do not merely provide opportunities for exercise; they also structure participation through repeated interaction, group routines, and shared identities (5). For that reason, the impact of sports clubs on happiness may not be reducible to “doing more exercise.” Rather, it may depend on whether club membership is translated into active participation, regular engagement, and embeddedness in a stable social setting. Recent studies support this possibility. Physical activity in social settings appears to generate happiness benefits partly because of processes such as social identification, not simply because people are physically active more often (6). Likewise, participation in sports clubs has been associated with stronger health behaviors and better perceived health outcomes, suggesting that clubs matter as organized settings that sustain activity and social involvement over time (7).

However, an important question remains unresolved in the existing literature. Previous studies have shown that physical activity and organized sport settings are associated with mental health, happiness, and social outcomes (1, 2, 8). Yet less is known about whether the happiness advantage of sports clubs is linked merely to formal membership or to actual participation in club-based activities. This issue is important because sports clubs are not only organizational settings but also contexts in which individuals may engage more fully in physical activity. This study therefore examines whether sports club participation (SCP) is associated with happiness and whether this association depends on actual participation rather than formal membership alone. Rather than treating club participation as a simple binary condition, the analysis distinguishes among non-members, inactive members, and active members, thereby separating nominal affiliation from substantive engagement. The central question is whether happiness is associated merely with belonging to a sports club, or with actively participating in it and engaging more fully in physical activity. By addressing this question, the study contributes to the literature in two ways. First, it shifts attention from broadly defined physical activity to organized SCP, highlighting the importance of distinguishing formal membership from active participation within sport organizations. Second, it examines whether the association between SCP and happiness is connected to behavioral engagement in physical activity, thereby moving beyond a simple membership-based explanation and providing a more nuanced account of how organized sport participation is associated with happiness.

## 2 Theoretical framework and hypotheses

### 2.1 SCP and happiness

Existing research has shown that physical activity is positively associated with happiness, mental health, and broader social outcomes (8), but recent studies also suggest that the benefits of organized sport may depend on the quality of participation rather than on mere affiliation alone (9). In organized physical activity settings, social identification and meaningful involvement appear to matter more for happiness than simple participation frequency or nominal group attachment. This implies that sports clubs should not be understood merely as formal memberships. Rather, they function as organized social settings in which repeated interaction, stable routines, and behavioral commitment may be translated into psychological gains. From this perspective, active participation is likely to be more beneficial for happiness than inactive or symbolic membership, because only active members are more fully embedded in the activities and social relations of the club (10).

Building on this logic, the present study distinguishes among non-members, inactive members, and active members. This distinction makes it possible to separate organizational belonging from actual engagement. If the psychological value of sports clubs derives primarily from sustained participation rather than nominal affiliation, then active members should report higher happiness than non-members, whereas inactive members are expected to show a weaker or even non-significant association. Accordingly, we propose:

H1: Active SCP is positively associated with happiness, whereas inactive membership shows a weaker or non-significant association.

### 2.2 SCP and physical activity

Sports clubs are not only social organizations; they are also practical settings that structure and sustain physical activity (11). Recent evidence indicates that participation in sports clubs is associated with more frequent weekly physical activity, healthier behaviors, and better perceived health outcomes (12, 13). These findings suggest that clubs can lower the barriers to participation by providing regular activity schedules, social support, behavioral norms, and opportunities for repeated engagement. In this sense, sports clubs may provide organizational settings in which individuals have more opportunities to engage in regular and sustained physical activity.

In the present study, this behavioral dimension is examined through two related but non-redundant forms of physical activity: leisure-time sports participation (LTSP) and venue-based exercise (VBE). LTSP refers to the frequency of sports or exercise participation during leisure time and captures the routine, frequency-based dimension of physical activity. VBE refers to the tendency to exercise in dedicated sports venues or gyms and captures the setting-oriented dimension of physical activity. Thus, LTSP emphasizes how often individuals engage in sports or exercise during leisure time, whereas VBE emphasizes whether such exercise takes place in dedicated sports or fitness settings. Because sports clubs may provide both opportunities for regular LTSP and access to more structured exercise settings, higher levels of SCP, especially active membership, are expected to be associated with higher levels of both LTSP and VBE. Therefore, we propose:

H2: SCP is positively associated with both LTSP and VBE, and this association is stronger for active members than for inactive members.

### 2.3 Physical activity, happiness, and the indirect pathways

Physical activity has been widely associated with happiness and mental health, but recent studies also suggest that different activity contexts may not be equally related to these outcomes (14, 15). In particular, LTSP has been shown to be positively associated with happiness, health, and mood, suggesting that activity embedded in everyday life may be especially relevant to happiness (3, 16). At the same time, VBE may represent a more structured form of activity that takes place in dedicated sports or fitness settings. Although LTSP and VBE both belong to the broader domain of physical activity, they capture different behavioral dimensions: LTSP emphasizes the frequency of LTSP, whereas VBE emphasizes the setting in which exercise is undertaken.

Based on this reasoning, physical activity may help explain the association between active SCP and happiness. Active SCP may be linked to higher happiness partly because active members are more likely to engage in both LTSP and VBE. However, because LTSP and VBE are measured using different question formats and capture different dimensions of physical activity, this study does not treat them as fully equivalent or directly interchangeable indicators. Instead, they are examined as two related but distinct indirect pathways. Accordingly, the present study proposes the following hypotheses:

H3: Both LTSP and VBE are positively associated with happiness.

H4: LTSP and VBE statistically mediate the association between active SCP and happiness.

### 2.4 Summary of the theoretical framework

**Figure 1:**
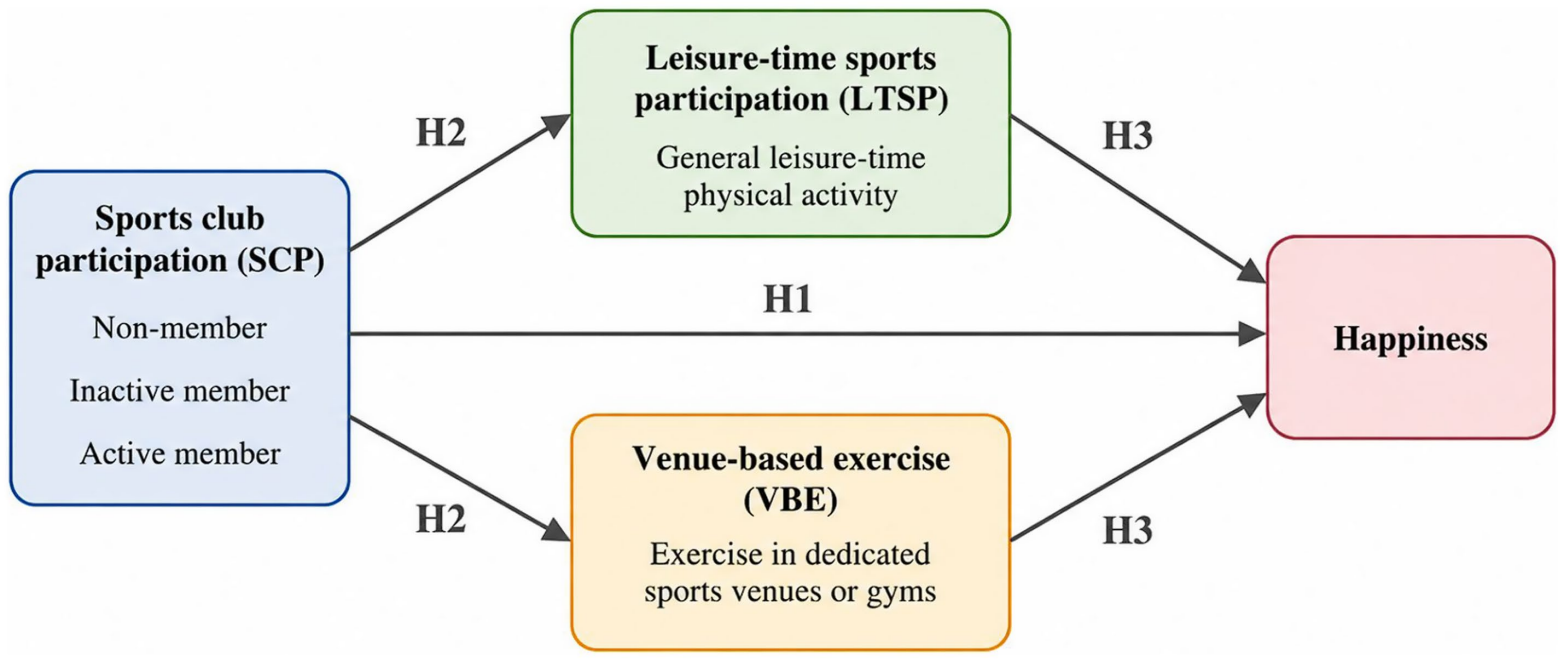
Theoretical framework.

As shown in Figure 1, the theoretical framework of this study conceptualizes the relationship among SCP, physical activity, and happiness as a process in which organizational participation is linked to behavioral engagement and happiness. The key distinction is not simply between members and non-members, but between nominal membership and active participation. Sports clubs may matter because active participation is more likely to be associated with repeated engagement in physical activity, including LTSP and VBE. In this framework, LTSP and VBE are treated as two related but distinct indirect pathways through which active SCP may be associated with happiness. This framework provides the basis for the empirical analyses that follow.

## 3 Materials and methods

### 3.1 Data source

This study draws on data from the 2023 Chinese General Social Survey (CGSS 2023), a nationwide social survey designed to capture a wide range of individual demographic characteristics, social attitudes, and behavioral patterns in China. The CGSS is widely used in social science research because of its broad population coverage and rich information on respondents ‘socioeconomic status, health, lifestyle, and happiness. These features make it particularly suitable for examining the relationship between SCP, physical activity, and happiness. After excluding observations with missing values on the dependent variable, key independent variables, mediators, and control variables, the final analytical sample consisted of 5,465 respondents.

### 3.2 Dependent variable

The dependent variable in this study is happiness. It is measured using a single self-reported item from the CGSS asking respondents: “Overall, do you think your life is happy?” Responses are coded on a five-point ordinal scale, ranging from 1 (very unhappy) to 5 (very happy), with higher scores indicating higher happiness. This item is used as a global self-assessment of happiness in a large-scale social survey rather than as a multi-item psychometric scale. Although single-item measures cannot assess internal consistency, previous research has shown that single-item happiness or global well-being measures can provide valid and useful indicators in large survey contexts (17–19). Therefore, this study interprets the outcome as general happiness, namely respondents’ overall perceived happiness status, rather than as a comprehensive multidimensional measure of subjective well-being. This measurement focuses on overall happiness and does not separately capture affective well-being, life satisfaction, or psychological flourishing. In the main analysis, happiness is treated as a quasi-continuous variable and estimated using OLS regression with robust standard errors. As a robustness check, it is further modeled as an ordered outcome using ordered logit regression.

### 3.3 Independent variable

The key independent variable is SCP. Respondents were asked whether they were members of a sports club and, if so, whether they actively participated in its activities. Based on their responses, this variable was coded into three categories: 0 = non-member, 1 = inactive member, and 2 = active member. Higher values indicate a greater level of actual involvement in sports club activities. In the regression models, non-members serve as the reference group.

### 3.4 Mediating variables

This study includes two mediating variables to capture two related but non-redundant dimensions of physical activity. LTSP is measured using an item from the CGSS leisure activity module, which asks respondents whether they had engaged in a series of activities during their spare time over the past year. Within this module, respondents were asked how often they participated in sports or exercise. The original responses ranged from “every day,” “several times a week,” “several times a month,” “several times a year or less,” to “never.” The variable was recoded so that higher values indicate more frequent participation in sports or exercise during leisure time. Therefore, LTSP captures the frequency-based and leisure-oriented dimension of physical activity.

VBE is measured using a separate self-reported item asking respondents whether the statement “I often go to dedicated sports venues or gyms to exercise” fits their lifestyle or actual situation. The original responses ranged from “fits very well” to “does not fit at all.” This variable was also recoded so that higher values indicate a stronger tendency to exercise in dedicated sports or fitness settings. Unlike LTSP, which is embedded in a broader leisure activity module, VBE captures a more structured and setting-oriented dimension of physical activity. It should therefore be understood as an indicator of exercise in dedicated venues or gyms, rather than as a direct measure of exercise intensity, duration, or motivation.

Although both variables reflect physical activity, they differ in their survey context and measurement focus. LTSP emphasizes how frequently respondents engage in sports or exercise as part of their leisure-time activities, whereas VBE emphasizes whether respondents tend to exercise in dedicated sports or fitness settings. Including both variables in the mediation analysis allows this study to distinguish between broader LTSP and more structured, VBE. To assess their potential statistical overlap, we further calculated the Pearson correlation between LTSP and VBE. The correlation was positive but modest (*r* = 0.271, *p* < 0.001), suggesting that the two variables are related but not statistically redundant. Multicollinearity diagnostics also showed low VIF values for LTSP (VIF = 1.23) and VBE (VIF = 1.20), with a mean VIF of 1.29 in the full model. These results support retaining LTSP and VBE as two related but distinct mediating variables.

### 3.5 Control variables

To reduce potential omitted-variable bias, a set of control variables is included in all models. These variables cover respondents’ socioeconomic background, demographic characteristics, and health status. Specifically, the controls include family economic status, place of residence, gender, age, education, marital status, political status, ethnicity, and self-rated physical health. Among these, gender, marital status, political status, and ethnicity are coded as binary variables. Place of residence is a four-category variable, distinguishing urban center, urban fringe or urban–rural fringe, town, and rural area; in the regression models, it is entered as a set of dummy variables, with urban center as the reference category. Age, family economic status, education, and self-rated physical health are treated as continuous or quasi-continuous variables.

### 3.6 Analytical strategy

The empirical analysis was conducted in three steps. First, the study estimated a series of ordinary least squares (OLS) regression models with robust standard errors to examine the associations among SCP, physical activity, and happiness. Given the imbalance in SCP categories, the OLS models were treated as baseline associational models rather than as standalone evidence. The analysis first examined whether SCP was associated with happiness. LTSP and VBE were then added to the happiness model to assess whether the coefficient of active SCP changed after these two physical activity variables were included. Additional models were estimated to examine the associations between SCP and the two mediators. This stepwise strategy made it possible to distinguish the total association between SCP and happiness from the remaining direct association after LTSP and VBE were taken into account. Because happiness was measured on a five-point ordered scale, the main models were further re-estimated using ordered logit regression as a robustness check to examine whether the substantive findings depended on treating happiness as a quasi-continuous variable.

Second, to address potential observable selection differences, the study employed propensity score matching (PSM) as an additional robustness check. In this stage, active club members were defined as the treatment group and non-members as the control group. Propensity scores were estimated based on respondents’ gender, age, education, marital status, political status, ethnicity, physical health, family economic status, and place of residence. Several matching methods were implemented, including nearest-neighbor matching, caliper matching, radius matching, and kernel matching, all under common support. The consistency of the average treatment effect on the treated (ATT) across these matching specifications was used to examine whether the positive association between active SCP and happiness remained after improving balance in observed covariates. However, because PSM can only adjust for observed characteristics, it should be interpreted as a robustness check rather than as evidence of causal identification.

Third, the study conducted a parallel mediation analysis using a bootstrap procedure to examine whether LTSP and VBE statistically mediated the association between active SCP and happiness. SCP was represented by two dummy variables, with non-members serving as the reference group. The mediation model simultaneously estimated the total effect, the indirect effect through LTSP, the indirect effect through VBE, the total indirect effect, and the remaining direct effect. The mediation analysis was based on 5,000 bootstrap replications, which provides a more reliable test of indirect effects without relying on normality assumptions. Because the data are cross-sectional, the mediation results should be interpreted as statistical indirect associations rather than as verified causal mechanisms.

## 4 Results

### 4.1 Descriptive results

Table 1 presents the descriptive statistics of the main variables. The dependent variable, happiness, has a mean of 3.899 (SD = 0.847), indicating a moderately high level of self-reported happiness in the sample. The key independent variable, SCP, is divided into three categories: non-members, inactive members, and active members. The descriptive results show a pronounced imbalance in SCP categories. Among the 5,465 respondents, 5,040 were non-members (92.2%), 136 were inactive members (2.5%), and 289 were active members (5.3%). This imbalance reflects the relatively low prevalence of sports club participation in the survey population. Because active members may differ systematically from non-members in observed background characteristics, the study further uses PSM as a robustness check to improve comparability between active members and observationally similar non-members. The two mediators, LTSP and VBE, have mean values of 2.569 (SD = 1.619) and 1.483 (SD = 0.757), respectively, indicating that physical activity is present but not highly frequent overall. The average age of the sample is 54.515 years, while the mean levels of physical health, economic status, and education are 3.351, 2.558, and 3.084, respectively. In addition, 44.8% of respondents are male, 71.4% are married, 84.1% are mass members, and 90.7% belong to the Han ethnic group. Regarding place of residence, 25.2% live in urban centers, 16.8% in urban fringe areas, 10.2% in towns, and 47.8% in rural areas.

**Table 1:** Descriptive statistics of the sample variables.

Variables	*N*	Mean	SD	Min	Max
Happiness	5,465	3.899	0.847	1	5
SCP					
Non-member	5,465	0.922	0.268	0	1
Inactive member	5,465	0.025	0.154	0	1
Active member	5,465	0.053	0.225	0	1
LTSP	5,465	2.569	1.619	1	5
VBE	5,465	1.483	0.757	1	4
Age	5,465	54.515	16.33	18	94
Physical health	5,465	3.351	1.141	1	5
Economic status	5,465	2.558	0.737	1	5
Education	5,465	3.084	1.249	1	5
Male	5,465	0.448	0.497	0	1
Married	5,465	0.714	0.452	0	1
Mass	5,465	0.841	0.366	0	1
Han ethnicity	5,465	0.907	0.29	0	1
Place of residence					
Urban center	5,465	0.252	0.434	0	1
Urban fringe	5,465	0.168	0.374	0	1
Town	5,465	0.102	0.303	0	1
Rural area	5,465	0.478	0.500	0	1

### 4.2 OLS regression results

Table 2 reports the OLS regression results for the relationships among SCP, happiness, LTSP, and VBE. Model 1 includes only the control variables and serves as the baseline model for happiness. Model 2 adds SCP to examine its association with happiness. The results show that, compared with non-members, active members reported significantly higher happiness (*β* = 0.151, *p* < 0.001), whereas inactive members did not differ significantly from non-members. This suggests that the positive association with happiness is concentrated among those who actively participate in sports clubs rather than those who merely hold membership. This finding supports H1, which proposed that active SCP would be positively associated with happiness, whereas inactive membership would show a weaker or non-significant association.

**Table 2:** OLS regression results for happiness, LTSP, and VBE (*N* = 5,465).

Variables	Model 1	Model 2	Model 3	Model 4 (LTSP)	Model 5 (VBE)
Inactive member		0.062 (0.059)	0.042 (0.059)	0.332** (0.117)	0.167* (0.072)
Active member		0.151*** (0.043)	0.098* (0.043)	0.874*** (0.088)	0.448*** (0.058)
LTSP			0.040*** (0.008)		
VBE			0.039** (0.014)		
Economic status	0.232*** (0.018)	0.230*** (0.018)	0.225*** (0.018)	0.047 (0.030)	0.063*** (0.013)
Urban fringe	0.106** (0.032)	0.106** (0.032)	0.117*** (0.032)	−0.146* (0.066)	−0.143*** (0.033)
Town	0.122** (0.036)	0.123** (0.036)	0.137*** (0.036)	−0.200** (0.073)	−0.151*** (0.039)
Rural area	0.088** (0.029)	0.090** (0.029)	0.127*** (0.029)	−0.587*** (0.059)	−0.328*** (0.027)
Gender	−0.053* (0.022)	−0.051* (0.022)	−0.062** (0.022)	0.151*** (0.041)	0.117*** (0.020)
Age	0.006*** (0.001)	0.006*** (0.001)	0.006*** (0.001)	0.009*** (0.002)	−0.006*** (0.001)
Education	0.046*** (0.012)	0.044*** (0.012)	0.032** (0.012)	0.277*** (0.022)	0.036*** (0.010)
Marital status	0.079** (0.025)	0.080** (0.025)	0.084** (0.025)	−0.086 (0.045)	−0.028 (0.021)
Political affiliation	−0.107*** (0.027)	−0.099*** (0.027)	−0.090*** (0.027)	−0.184** (0.060)	−0.043 (0.032)
Ethnicity	−0.001 (0.040)	0.004 (0.040)	0.001 (0.039)	0.126 + (0.068)	−0.043 (0.036)
Physical health	0.151*** (0.011)	0.150*** (0.011)	0.143*** (0.011)	0.141*** (0.020)	0.034*** (0.009)
Constant	2.330*** (0.108)	2.319*** (0.108)	2.216*** (0.111)	0.965*** (0.197)	1.656*** (0.094)
R^2^	0.116	0.117	0.124	0.161	0.140

*p < 0.05, **p < 0.01, ***p < 0.001.

Model 3 further adds LTSP and VBE. Both variables were positively associated with happiness (LTSP: *β* = 0.040, *p* < 0.001; VBE: *β* = 0.039, *p* < 0.01), supporting H3. After LTSP and VBE were introduced, the coefficient for active membership declined from 0.151 to 0.098 while remaining statistically significant (*p* < 0.05). This pattern provides preliminary evidence that the association between active SCP and happiness may be partly linked to physical activity, although the formal mediation results are reported in Table 3. The magnitude of the coefficient should also be interpreted carefully. On a five-point happiness scale, the change from 0.151 to 0.098 indicates a statistically significant but modest difference in happiness. Therefore, the observed association should not be interpreted as a large individual-level change. Nevertheless, because SCP and physical activity are modifiable lifestyle-related behaviors, even modest associations may have population-level relevance when considered across broader community, school, university, or public health settings.

**Table 3:** Bootstrap results of the parallel mediation analysis.

Path	Coefficient (SE)	*z*	95% CI
Inactive member → LTSP → happiness	0.013** (0.005)	2.45	[0.003, 0.024]
Inactive member → VBE → happiness	0.006 (0.004)	1.66	[−0.001, 0.014]
Inactive member: total indirect effect	0.020** (0.007)	2.86	[0.006, 0.034]
Inactive member: direct effect	0.042 (0.059)	0.72	[−0.073, 0.158]
Inactive member: total effect	0.062 (0.059)	1.05	[−0.054, 0.178]
Active member → LTSP → happiness	0.035*** (0.007)	4.79	[0.021, 0.050]
Active member → VBE → happiness	0.017* (0.007)	2.52	[0.004, 0.031]
Active member: total indirect effect	0.053*** (0.010)	5.46	[0.034, 0.072]
Active member: direct effect	0.098* (0.043)	2.30	[0.014, 0.182]
Active member: total effect	0.151*** (0.043)	3.54	[0.067, 0.234]

*p < 0.05, **p < 0.01, ***p < 0.001.

Models 4 and 5 examine the associations between SCP and the two mediators. The results show that both inactive members and active members reported significantly higher levels of LTSP and VBE than non-members, but the coefficients were larger for active members. In particular, active membership was strongly associated with both LTSP (*β* = 0.874, *p* < 0.001) and VBE (*β* = 0.448, *p* < 0.001). This finding is consistent with H2, which predicted that SCP would be positively associated with both LTSP and VBE, with stronger associations for active members than for inactive members. Taken together, Table 2 suggests that active SCP is associated with higher happiness, that both LTSP and VBE are positively associated with happiness, and that active membership is more strongly linked to both forms of physical activity than inactive membership.

### 4.3 Robustness check using ordered logit models

Table 4 reports the ordered logit estimates as a robustness check for the main OLS results. Because happiness is measured on a five-point ordinal scale, this analysis examines whether the main findings remain consistent when happiness is treated as an ordered outcome rather than as a quasi-continuous variable. The results are substantively consistent with the OLS estimates. In the model without mediators, active membership was positively and significantly associated with happiness (*β* = 0.426, *p* < 0.001), whereas inactive membership was not statistically significant. This pattern provides further support for H1, which proposed that active SCP would be positively associated with happiness, while inactive membership would show a weaker or non-significant association.

**Table 4:** Ordered logit regression results for happiness (*N* = 5,465).

Variables	Model 6	Model 7	Model 8	Model 9
Inactive member	0.146 (0.148)	0.125 (0.149)	0.111 (0.148)	0.098 (0.149)
Active member	0.426*** (0.118)	0.370** (0.120)	0.332** (0.118)	0.299* (0.120)
LTSP		0.120*** (0.037)		0.083* (0.038)
VBE			0.110*** (0.019)	0.104*** (0.019)
Control Variable	YES	YES	YES	YES
Pseudo *R*^2^	0.0479	0.0487	0.0509	0.0512

*p < 0.05, **p < 0.01, ***p < 0.001.

After LTSP and VBE were added separately and jointly, the coefficient for active membership declined from 0.426 to 0.370, 0.332, and 0.299, respectively, but remained statistically significant across all specifications. In addition, both LTSP and VBE were positively and significantly associated with happiness when entered separately, and both remained significant in the full model. These results provide further support for H3, which proposed that both LTSP and VBE would be positively associated with happiness. The decline in the coefficient for active membership after the inclusion of LTSP and VBE is also consistent with the expectation that the association between active SCP and happiness is partly linked to physical activity, although the formal mediation analysis is reported in Table 3. Overall, the ordered logit results strengthen the robustness of the main findings by showing that the conclusions are not dependent on treating happiness as a continuous outcome.

### 4.4 Propensity score matching results

Table 5 reports the PSM results comparing active members with non-members. Given the imbalance in SCP categories, the PSM analysis was conducted as a robustness check to examine whether the positive association between active membership and happiness remained after improving comparability on observed covariates. Across different matching methods, the estimated ATT remained positive and statistically significant, ranging from 0.189 to 0.210. This indicates that active members reported higher happiness than matched non-members with similar observed characteristics.

**Table 5:** PSM results under different matching methods.

Matching method	ATT	SE	*t*	Mean Bias	Ps *R*^2^
1:1 NN	0.189***	0.061	3.12	3.6	0.004
1:2 NN	0.210***	0.055	3.83	3.2	0.003
Radius matching	0.199***	0.045	4.42	9.0	0.012
Kernel matching	0.194***	0.045	4.31	8.1	0.010

NN = nearest-neighbor matching; Radius = radius matching with caliper = 0.05. *p < 0.05, **p < 0.01, ***p < 0.001.

The last two columns report balance statistics after matching. Mean Bias refers to the average standardized covariate imbalance between the treatment and control groups, with smaller values indicating better balance. Ps *R*^2^ reflects the extent to which the observed covariates still predict treatment assignment after matching, with values closer to zero indicating better comparability. Among the matching specifications, the 1:2 nearest-neighbor matching achieved the lowest Mean Bias (3.2) and the lowest Ps *R*^2^ (0.003), suggesting improved covariate balance after matching. These results are consistent with the OLS findings and provide additional robustness support for H1, which proposed that active SCP would be positively associated with happiness.

**Figure 2:**
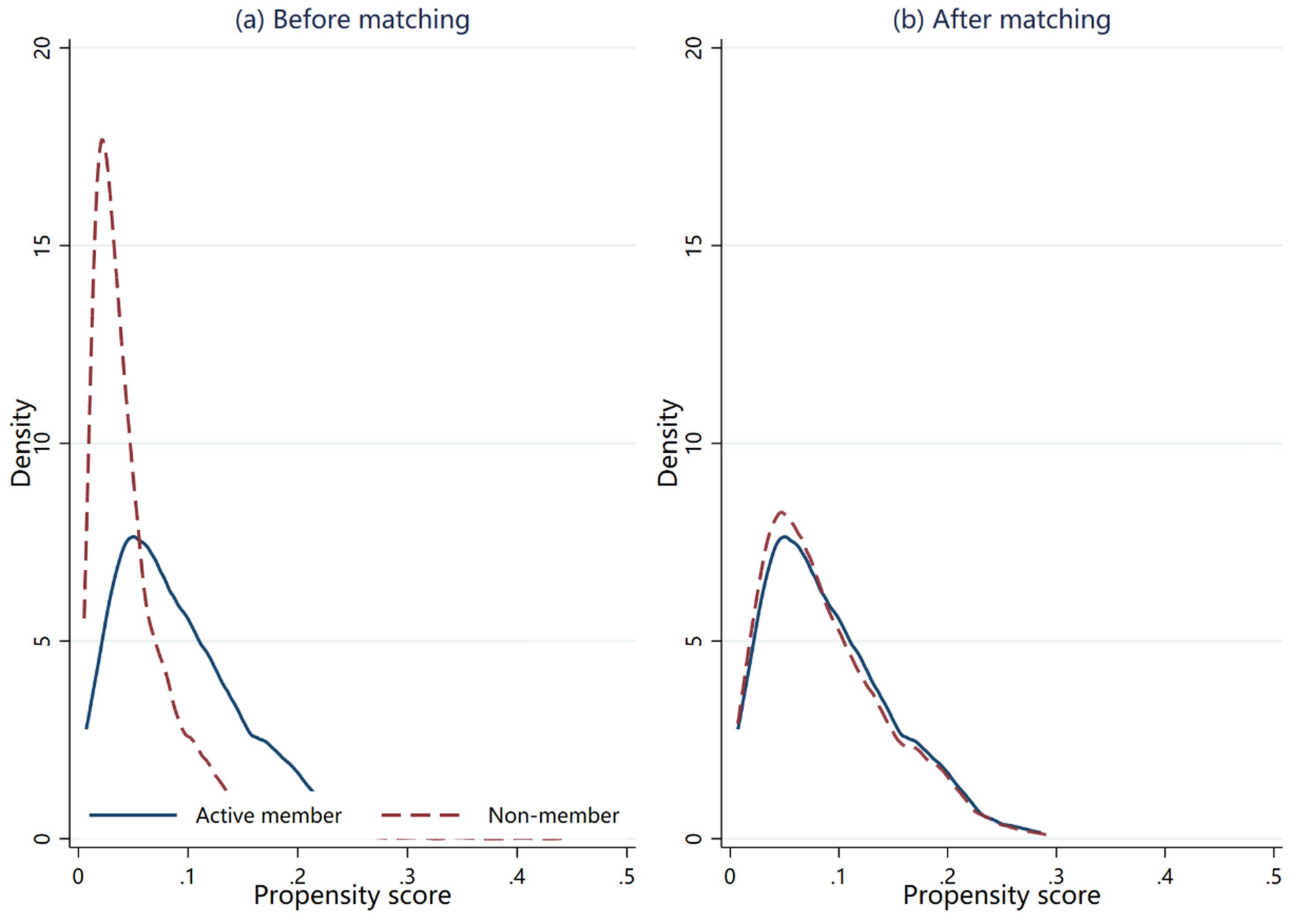
Propensity score distributions before and after matching.

Figure 2 further illustrates the propensity score distributions of active members and non-members before and after matching. Before matching, the two groups showed visible differences in their propensity score distributions, suggesting observable selection into active club participation. After matching, the density curves overlapped more closely within the region of common support, indicating improved comparability between the matched groups. However, because PSM only adjusts for observed covariates, these results should be interpreted as robustness evidence rather than as proof of a causal effect.

### 4.5 Mediation effects

Table 3 presents the bootstrap results of the parallel mediation analysis for happiness. For inactive members, the total effect on happiness was not statistically significant. Although the indirect effect through LTSP and the total indirect effect were statistically significant, the VBE path was not significant because its 95% confidence interval included zero. Therefore, the results for inactive members are interpreted cautiously as limited evidence of an indirect association, mainly through LTSP. Because the total effect of inactive membership was not significant, proportional mediation sizes are not reported for this group. For active members, the results provide clearer evidence of partial mediation. The total effect of active membership on happiness was positive and statistically significant, and both indirect paths through LTSP and VBE were significant. The total indirect effect accounted for 34.87% of the total association between active membership and happiness, including 23.33% through LTSP and 11.54% through VBE. These findings support H4 by showing that LTSP and VBE partially mediate the association between active SCP and happiness.

## 5 Discussion

### 5.1 Active participation matters more than membership

A first key finding of this study is that the association between SCP and happiness depends on actual participation rather than formal membership alone. In our models, inactive membership was not significantly associated with happiness, whereas active membership remained positively associated with happiness across the main OLS models, the ordered logit robustness checks, and the PSM analysis. This result both confirms and refines prior research. Existing studies have consistently shown that physical activity is positively related to happiness, mental health, and broader happiness outcomes, including in the Chinese context (16), but much of that literature treats participation rather broadly and pays less attention to differences in the intensity or quality of organizational involvement (20, 21). Our findings suggest that the happiness advantage of sports clubs is not attached to organizational affiliation itself, but is observed mainly among those who actively participate in club-based activities.

This distinction is important because it shows that SCP should not be understood merely as a formal membership status. The non-significant result for inactive membership indicates that nominal membership alone does not provide a detectable happiness advantage in this sample. Therefore, rather than theorizing detailed psychological or social mechanisms for inactive members, a more cautious interpretation is that formal affiliation without active engagement is not significantly associated with happiness. By distinguishing among non-members, inactive members, and active members, this study extends previous research by showing that the key empirical distinction lies not simply in whether individuals belong to a sports club, but in whether they are actively involved in club-based participation. This finding is consistent with recent evidence suggesting that continued and meaningful participation in sport settings is linked to more favorable happiness and health-related outcomes (22, 23).

### 5.2 Physical activity as an indirect pathway

A second major finding is that physical activity serves as an important indirect pathway linking active SCP and happiness. In our models, both LTSP and VBE were positively associated with happiness, and the coefficient for active membership declined after these two mediators were introduced. This pattern suggests that part of the positive association between active SCP and happiness is statistically linked to greater engagement in physical activity. This result is consistent with a growing body of research showing that sport and exercise are positively associated with happiness, mental health, and broader social outcomes (1). At the same time, our findings provide a more specific account of SCP. Sports clubs are not only formal organizations, but also settings in which active members may engage more regularly in physical activity. In this sense, physical activity appears to be one behavioral pathway through which active club participation is associated with happiness. The fact that the direct effect of active membership remained significant after LTSP and VBE were included further suggests that physical activity is an important, but not exclusive, pathway linking active SCP to happiness.

### 5.3 Practical implications

The findings of this study suggest that efforts to promote happiness through sports clubs may need to focus not only on increasing formal membership, but also on supporting active and sustained participation. Since the positive association with happiness was observed mainly among active members rather than inactive members, community organizations, schools, universities, and local governments may benefit from paying greater attention to participation quality, continuity, and engagement rather than enrollment alone. In addition, the mediation results suggest that active SCP is partly linked to happiness through LTSP and VBE. Although the indirect association through LTSP was numerically larger in the present models, this pattern should be interpreted cautiously because LTSP and VBE were measured using different question formats and capture different dimensions of physical activity. Therefore, sports promotion strategies should not be understood as favoring one activity form over another. Rather, they may consider how to support both routine LTSP and access to more structured, VBE opportunities. In practical terms, low-threshold, regularly organized, and socially accessible activities may help individuals maintain physical activity in everyday life, but future intervention studies are needed to determine whether such strategies can lead to measurable improvements in happiness.

### 5.4 Limitations

This study has several limitations. First, the analysis is based on cross-sectional data, which limits causal interpretation. Although OLS models, ordered logit models, PSM, and bootstrap mediation analysis were used to examine the robustness of the findings, these methods cannot establish temporal ordering or fully rule out reverse causality. Individuals who are already happier, healthier, or more socially active may be more likely to join sports clubs, become active members, and engage in physical activity. Therefore, the findings should be interpreted as associational evidence rather than as proof that SCP causes higher happiness. Future longitudinal or experimental studies are needed to examine whether changes in sports club participation precede changes in happiness.

Second, happiness was measured using a single self-reported item. Although single-item happiness measures are commonly used in large-scale social surveys and can provide a useful global assessment, they cannot be tested for internal consistency and may contain measurement error. In addition, this measure captures general self-reported happiness rather than a comprehensive multidimensional measure of subjective well-being. It does not separately distinguish affective well-being, life satisfaction, or psychological flourishing. Future research using multi-item well-being measures could examine whether the findings remain consistent across different dimensions of well-being.

Third, LTSP and VBE are measured using different question formats and capture different dimensions of physical activity. LTSP reflects the frequency of sports or exercise during leisure time, whereas VBE reflects the tendency to exercise in dedicated sports venues or gyms. Although the correlation and VIF diagnostics suggest that the two variables are related but not statistically redundant, their coefficients should not be interpreted as directly comparable indicators of the same construct. Therefore, this study avoids drawing strong conclusions about whether one form of physical activity is more effective than the other. Future studies with more refined and comparable measures of activity frequency, duration, intensity, setting, and quality would help clarify the relative roles of different forms of physical activity.

Fourth, the study does not directly measure the social dimension of sports club participation. Sports clubs may provide opportunities for interaction, belonging, peer support, and social identification, but the available CGSS data do not include direct measures of these relational mechanisms. As a result, this study cannot determine whether the association between active SCP and happiness is driven by the social characteristics of sports clubs, by increased physical activity, or by both. Future research should incorporate measures of social capital, group belonging, interpersonal interaction, and peer support to examine the social pathways of sports club participation more directly.

Fifth, the distribution of SCP categories is highly imbalanced. Most respondents were non-members, while inactive and active members accounted for relatively small proportions of the sample. This imbalance may affect the stability and precision of subgroup estimates, especially for inactive members. Although robustness checks were conducted, including ordered logit models, PSM, and bootstrap mediation analysis, these methods cannot fully eliminate concerns related to sparse subgroup observations or unobserved selection. Therefore, the findings for inactive membership should be interpreted with particular caution. Future studies with larger samples of sports club members are needed to further examine differences between inactive and active participation.

## 6 Conclusion

This study examined the association between SCP and happiness, with particular attention to the statistical indirect pathways through LTSP and VBE. The results lead to two main conclusions. First, the positive association between SCP and happiness is concentrated among active members, whereas inactive membership does not show a significant association with happiness. This suggests that the relevance of sports clubs for happiness is not attached to formal affiliation alone, but is observed mainly when membership involves active participation. Second, LTSP and VBE both serve as significant indirect pathways linking active SCP and happiness. This indicates that the association between active club participation and happiness is partly connected to greater engagement in physical activity. Taken together, these findings highlight the importance of distinguishing nominal membership from active participation in sports clubs and suggest that organized sport settings may be relevant to happiness when they are accompanied by sustained behavioral engagement. Given the cross-sectional design, these conclusions should be interpreted as associational rather than causal evidence.

## Data Availability

Publicly available datasets were analyzed in this study. This data can be found at: the data used in this study are from the 2023 wave of the Chinese General Social Survey (CGSS), which is publicly available at the CGSS data repository (http://cgss.ruc.edu.cn/). Researchers can access the dataset by registering on the official website.
